# How to Teach/Learn Praecox Feeling? Through Phenomenology to Medical Education

**DOI:** 10.3389/fpsyt.2022.819305

**Published:** 2022-03-18

**Authors:** Tudi Gozé

**Affiliations:** ^1^Department of Psychiatry, Psychotherapies, Art-therapy, Toulouse University Hospital, Toulouse, France; ^2^Equipe de Recherche sur les Rationalités Philosophiques et les Savoirs - EA3051, Université de Toulouse - Jean Jaurès, Toulouse, France

**Keywords:** medical education, schizophrenia, phenomenology, Praecox Feeling, diagnostic

## Abstract

**Background:**

The Praecox Feeling (PF) refers to a classical psychopathological concept describing the specific experience of bizarreness arising in the encounter with a person living with schizophrenia spectrum disorders (SSDs). Some studies have shown that experienced psychiatrists take advantage of this experience to perform accurate and rapid diagnostic expertise. It would seem that PF is not contradictory with an operationalized diagnostic approach, but that the PF would intervene at a more tacit level of medical judgment. However, the articulation between the implicit and explicit levels of the psychiatrist's experience in the situation of medical judgment remains little studied, even though it is of crucial importance for structuring the teaching of clinical psychiatry to mental health practitioners. Can diagnostic intuition be learned? Is this experience a kind of “gift” that some may or may not have? Does the PF refer to medical expertise?

**Methods:**

To unfold the complexity of his questions this article proposes to conduct an historical, epistemological and phenomenological analysis of the PF.

**Results:**

We will first conduct a presentation of historical descriptions of the PF understood as a sensation, intuition and experience, alongside the evolution of the concept of schizophrenia. Then, the article proposes an original phenomenological modelization of the temporal unfolding of the PF.

**Discussion:**

The phenomenological conceptualization, informed from empirical evidence will try to account for the paradox of the PF as both lived evidence and indescribable experience. PF will be described as a complex cognitive and embodied process based upon ante-predicative aesthetic sensing which is secondly apprehended as perceptible evidence thanks to clinical typification. This conceptualization relying on Husserl manuscript on intersubjectivity will help to demystify its experiential structure and discuss its relevance for medical education.

## Introduction

Schizophrenia is one of the most frequent and invaliding mental conditions. Since there is no valid biomarker of schizophrenia, clinical expertise remains referential for diagnostic decision-making. Despite a process of regular improvement of international disease classifications (DSM and ICD systems) to achieve the twin goals of good interjudge reliability and validity. It is now widely recognized that neither of these objectives is being met in a way that satisfies the legitimate demands of the population for early and accurate diagnosis. This observation is often made very early on by young doctors embarking on their specialization in psychiatry, which does not fail to produce a discouraging effect. It is likely that part of the reluctance of medical students to specialize in psychiatry is due to the lack of scientific and clinical consistency. Several authors have hypothesized a progressive and not intentional loss of psychopathological knowledge since the widespread use of the DSM system in psychiatry teaching (1, 2).

In this article I would like to advocate an approach to medical pedagogy that supports the development of the student's psychopathological thinking, in particular by encouraging the student's ability to be attentive to his or her own experience and reasoning process during the encounter with the patient. I will try to show that such an approach not only has scientific validity, but also leads to a more reliable and valid diagnosis in the case of schizophrenia. For this I would take the paradigmatic example of Praecox Feeling (PF). The PF refers to a classical psychopathological concept coined by Rümke (3), describing the specific experience of bizarreness arising in the encounter with a person living with schizophrenia spectrum disorders (SSDs). Some historical and contemporary studies have shown that experienced psychiatrists take advantage of this experience to perform accurate and rapid diagnostic decision making. It has also been suggested that the PF is not contradictory with an operationalized diagnostic approach, but that the PF would intervene at a more tacit level of medical judgment (4). However, the articulation between the implicit and explicit levels of the psychiatrist's experience in the situation of medical judgment remains little studied, even though it is of crucial importance for structuring the teaching of clinical psychiatry to mental health practitioners. By conducting a phenomenological analysis of this specific experience, I will attempt to demystify this notion, which may appear as intuition or clinical flair, which would not fail to raise a criticism of arbitrariness.

I will first conduct a presentation of historical descriptions of the PF alongside the evolution of the conceptual history of schizophrenia. Then, the article proposes an original phenomenological modelization of the temporal unfolding of the PF and possible implication for medical pedagogy.

## A Conceptual History of the Praecox Feeling

Praecox Feeling is a highly ambiguous notion in the conceptual history of schizophrenia. The historical and conceptual interconnections between the notion of schizophrenia and PF were recently discussed in greater detail by Pallagrosi and Fonzi (5) and myself (6). From this story, it is important to retain that PF was first described by Rümke (3) as an ineffable experience arising in the psychiatrist during an interview with a person living with schizophrenia. Rümke asserts that this lived experience is accurate in differentiating true schizophrenias from non-schizophrenic forms of psychosis. This assertion is historically embedded in the criticism of the neo-Kraepelinians with whom Rümke is affiliated, addressing the definition of schizophrenia proposed by Bleuler (7), which they consider too broad and unspecific. The history of the PF is thus intimately linked to the conceptual history of schizophrenia (8).

Rümke claimed that the diagnosis of schizophrenia is often fairly quickly reached through a passive and unformalisable intuition. Rümke stated that it is remarkable that “it is rare for a clinician to be able to say exactly how he arrives at a diagnosis of schizophrenia” (3), the so-called positive symptoms being, in his view, non-specific and cannot explain the psychiatrist's diagnostic reasoning. If the symptoms, taken individually, are unspecific, together they appear as having something of a “schizophrenic color.” There is a specific atmosphere of the encounter that refers, in his view, to the inability to come into empathic contact with the patient personality as a whole (9). Even if one might think that empathic distance is a barrier to diagnosis, Rümke suggests that it is precisely this atmosphere that is specific to the clinical core of schizophrenia.

The notion of clinical core and its intuitive capture was further developed by the French-polish psychiatrist Eugene Minkowski. Inspired by philosophers Henri Bergson and Max Scheler, Minkowski substantially improved the Bleulerian conception of schizophrenic autism, and argued that it is possible to perform a direct recognition of what he called “the loss of vital contact with reality” (10). This ability is described as “diagnostic by penetration” (11). Ludwig Binswanger, the founder of phenomenological psychotherapy, highlighted in 1924 the possibility of diagnosing schizophrenia by feeling (*Gefühlsdiagnose*) through face-to-face interaction. He argued that the relationship between a doctor and a patient operates at a fundamentally different level than the objective perception of symptoms as “the impression that there is a barrier that prevents me from uniting myself deeply with him” [(12), p. 136]. The idea of the breakdown of empathic contact, and thus of psychopathological understanding, is an idea that we owe to the father of modern psychopathology, Jaspers (13), for whom the incomprehensibility of the schizophrenic experience is a determining criterion of the diagnosis. After him, Wolfgang Blankenburg affirms that the psychiatrist's conscience is the “sensitive reagent” which allows to become aware of this incomprehensibility in order to make it a reliable clinical sign. The patient's “loss of natural evidence” leads him to a feeling of strangeness (or extraneation, *Entfremdung*). According to him, the psychiatrist feels this strangeness in contact with schizophrenia (extraneity, *Befremdung*) is a mirror of what the patient feels. He then insists that the doctor must open up (*aufschliessen*) to this extraneity in order to hope to understand the patient's experience (14).

The project to operationalize psychiatric diagnosis originated with concerns about the unreliability of the diagnosis of schizophrenia in the late 1970s (15) and led to the formulation of DSM III in 1980 led to the formulation of DSM-III in 1980. The ambition of this program was to enhance interrater reliability by the operationalization of diagnosis judgment and was heavily based on standardized tools for structure interview methods grounded in symptom checklists. These data were supposed to be context and observer independent (16, 17), focussing on third-person observation, so called “objective data,” which are assumed to be observer independent. This movement, which was supported internationally, tried to base psychiatry outside the first-person experience of the clinician, which was suspected of being arbitrary (so called subjectivity).

This is quite legitimate in view of the descriptive epidemiological objectives, methodological, and ethical constraints that the international psychiatric community gave itself in 1977 at the Sixth World Congress of Psychiatry held in Honolulu (Hawaii) and which led to the project of an operational and homogenization of classification of mental illness. Fifty years after this event, we can only deplore the confusion of these laudable objectives with the agenda of a biological and positivist psychiatry that has participated in confusing subjectivity (of the patient, of the psychiatrist) with subjectivism and arbitrariness. As a result, the PF was now considered too “subjective” and incompatible with the project of scientific psychiatry and disappeared from the psychiatric literature.

The “operational revolution” (18) has profoundly modified the teaching of psychiatric practice worldwide. While the DSM and ICD systems aimed to homogeneize the categories to allow for consistent and large-scale epidemiological and pharmacological studies, it turned out that these “manuals” were used to structure the teaching of medical students, young specialists and paramedical teams (1). The density of descriptive knowledge has thus become dangerously weakened. This loss of psychopathological culture was suspected by some influential scholars as a loss of clinical competencies. In 2013 preliminary studies to assess interjudge reliability of diagnostic categories with DSM 5 criteria had put forward an unsatisfactory level of reliability for schizophrenia (19, 20). This may suggest that the program of complexity reduction, operationalization and de-subjectivation of categories has not led to improved reliability of diagnostic judgment. On the other hand, we know that the validity of the diagnosis is an even greater problem in the absence of a consensual definition of schizophrenic illness and a reliable and operational biomarker (21). It then seems legitimate to ask what is the role of the clinician's subjectivity in the diagnostic process. Is it a confounding factor, or an indispensable ingredient, as Blankenburg says, “a sensitive reagent”?

Whereas, Rümke was the first to use the term, the idea of intuitive and immediate diagnosis belonged to a much longer discussion taking place in continental psychopathology in the nineteenth and early twentieth century. For example, Asperger wrote that “autistic behavior has its own particular flavor which is unmistakable for the experienced” [(22), p. 50]. Carp spoke of an “hysteria feeling” (23). Rapid decision and intuitive impressions also play a role in medical care. Some studies refers to “gut feeling” in nurse-patient relationship (24, 25), critical care (26), general practice (27), and obstetrics (28) as an experienced gained intuitive “knowledge” structuring, through critical thought, a deeply grounded knowledge base that can be applied in daily practice. These studies suggest that the “gut feeling” refers to a tacit perception of the distress experienced by the patient, which is “sensed” in an undefined way by the clinician, notably through non-verbal attitudes. All these authors, in psychiatry as well as in medicine, have insisted on the fact that direct intuition can only support the care process insofar as it is identified by the care-giver in his or her own experience, and subjected to critical reflection and consultation between professionals. Under these conditions, intuition can have great diagnostic, prognostic, or therapeutic value. How then can we think of the articulation, within the medical judgment, of the tacit dimension specific to PF, and the dimension of explicit research of diagnostic criteria? How do these two dimensions fit together and how could they lead to a reliable and scientifically consistent diagnostic decision making?

While it is legitimate to be highly skeptical of the validity and reliability of the PF-like experiences, there are now some arguments to document this experience. Very few empirical studies have explored the PF. There is some evidence that the PF has at least some clinical validity and that it still plays a role in diagnostic decision making in schizophrenia. Two studies investigated the sensitivity and specificity of PF compared to standardized diagnostic classifications with different methodologies (29, 30). The two studies, already old, showed very different results. Since then, no further studies have been attempted, so the results cannot be used as they stand. In addition it should be noted here that the assessment of the validity of PF as a diagnostic tool for schizophrenia is very delicate insofar as there is no gold standard against which to compare it. A second type of evidence concerns the prevalence of psychiatrists reporting to rely on PF in diagnostic decision making. Four studies were conducted with a comparable protocol in Germany in 1962 (31), in the USA in 1989 (32), in France in 2017 (33), and in Poland in 2019 (4). These studies indicate that PF is still used with an average rate of 86.8% (*N* = 1,874) of psychiatrists surveyed occasionally experiencing PF. We have observed a stability of the indicators since the 1960s until 2020s, without any significant variation between the countries studied. These results allowed us to develop the hypothesis that the teaching of criteriological methods as cardinal diagnostic skills for Evidence Based Medicine did not lead to any significant relegation of PF from routine diagnostic decision-making.

## On the Phenomenology of the Praecox Feeling

Phenomenological perspective in psychiatry, following Edmund Husserl's method, invites to thwart the confusion between what he called the “doxa,” which corresponds to the opinion that I hold on such and such an event or experience (that is, subjectivism), and the lived experience (phenomenon) after the “bracketing” (34) of its clothes of ideas and social convention. The phenomenological epoché ($\overset{'}{\varepsilon }\pi o\chi \dot{\eta }$) consists in the suspension of any judgment pertaining to a social construction or a position of being (35, 36) while remaining immersed in the experience. From this methodological point of view, it is precisely a question of thwarting all a priori on a lived experience, whether these apriori are scientific, political, social, psychological, in order to give an account only of the appearing of the lived experience to the consciousness. We would like to show in this article that Husserl's methodological and epistemological gesture remains relevant today in order to give an unprejudiced account of the way the psychiatrist's conscious experience unfolds in the situation of the schizophrenic encounter.

How phenomenology could help in giving the methodological resources to model PF in diagnostic judgement about schizophrenia? How the indisputable but ineffable atmosphere of bizarreness becomes a clinically perceptible sign? As I have argued elsewhere (37), the phenomenological description of the PF cannot be direct, and is a methodological challenge for psychiatric phenomenology itself. Indeed, as Rümke and Minkowski have indicated, PF refers primarily to the breakdown of empathic or affective contact with the person. It is therefore a description of an experience of incomprehension or strangeness that is most often described as ineffable, indescribable, or unnameable. Paradoxically, this experience is also described as a lived evidence, or a certainty that is formed very quickly in the clinician's experience. The paradox of the PF can thus be stated as follows: it is obvious and indescribable.

To describe the movement of the clinician's thinking during the interview, Müller-Suur (38) described the PF as an “indefinite un-understandability” of the patient experience. That is, it is initially experienced as a vague feeling of strangeness of the encounter. Further on, the clinician searches for disconfirming evidence, through a process of critical reflection, the incomprehensibility of the patient that initially struck the psychiatrist becomes definite and can serve as a reliable clinical manifestation. A very close concept can be found later on in the works of Schwartz and Wiggins (39). These authors have coined the concept of typification, inherited of Husserl's description of perceptual experience. Typification is neither arbitrary nor intuitive. Typification can be described as a basic and tacit perceptual processing that permits recognition of a form (*Gestalt*) under the condition of incomplete data giveness. For example, we do not need to have an overall view of a building for the partial perception of one of its facets (*Abschattung*) to immediately send us back to the idea (*eidos*) of the building and permit the anticipation of its hidden facets. Schwartz and Wiggins have argued that thanks to typification it is possible for a trained clinician to recognize in the first minutes of the encounter that a patient presents a certain *Gestalt* of personality. They claimed that typifications reveal the ideal-typical connections between independent signs to have an experience of the patient as a united whole (40, 41). The initial typification evolves along the interviewing process from a mainly tacit and elusive feeling to a more nuanced and specific impression. Schwartz and Wiggins argued that the typification is scientifically reliable only if it is based upon a dynamic circle of recognition and verification by the evidence-based criteria. This description of the “march” of clinical reasoning provides a more ecological account of how psychiatric thought is constructed. For example, in an emergency room interview we never explore all the symptoms of SCID, which would be necessary for a correct DSM diagnosis. Instead, our impressions, our intuitions, like the PF, guide us to move more quickly or insistently toward the symptoms that are felt to be relevant. This is the case with delusions or acoustic-verbal hallucinations. The scientific use of typifications requires that psychiatrists also doubt and reflect on their typifications and repeatedly test their interpretations by looking for additional components to prove or correct their typifications (42). This idea could suggest that PF is not opposed to a criteriological attitude in diagnosis, but that clinician's reasoning navigates constantly between a basic empathic, non-declarative or tacit experience, then the recognition of this experience as specifically relating to the schizophrenic quality of the encounter, then in a third stage, the submission of this experience to confirmation or disconfirmation with regard to objective clinical signs.

The problem of the phenomenal givenness (*how* is it given as an experience) of PF has already been the subject of much debate. The majority of contemporary psychiatric literature follows the concept of typification developed by Schwartz and Wiggins (39), which can be resumed as a tacit process of object consciousness. The PF understood as a typification ultimately leads to a predicative judgement. It is admittedly partly tacit to consciousness, but well within the scope of the perceptual intentional process—intentional in the phenomenological sense of being directed at something. This conceptualization certainly helped to legitimize PF as a medically valid experience. Nevertheless, it also brings about a theoretical impasse. The critique of typification comes from Husserl himself and his arguments against analogization. The most well-known argument is presented in the famous 5th *Cartesian meditation* (*Hua I*). In this text, Husserl questions the idea that the experience of others proceeds as to the perception of an object that can be typified as a whole, even though only some of its “facets” (*Abschattung*) is perceived. There is always an internal horizon to the object that is given in the perception, which allows one to have several perspectives on the same object and to anticipate the possible forms of this object so that the world remains continuous and reliable in its identity. With regard to the other, Husserl notes that there is an unfathomable reserve of otherness. One cannot “go around” the other to reveal all his/her facets. If one can understand others, it is by a process of apperception, where the gap of otherness is somehow crossed by analogy with one's own embodied experience.

At the beginning of twentieth century, the question of the experiential “nature” of empathy was much debated in the scientific and philosophical literature. Husserl discussed all his life the works of German psychologists (43, 44), on the status of analogy. He pointed out that empathy is not simply a question of attaching an “image” of one's embodied self to the appearing body of others to experience the other body as an embodied presence (45). If this were the case, one would see in others only avatars of oneself, the look-alikes responding to one's intentions. This is similar to the experience that is described by people with Capras syndrome (or delusions of doppelganger) but cannot account for ordinary experience of human encounter. The givenness of the other as an other is possible because one's corporeality is the matrix of appearance that itself contains a fundamental otherness (transcendence) (46, 47). It is because one makes the experience of exteriority (the surrounding world) by the means of one's living corporality and according to the *habitus* of one's body schema, that the appearing body of others is not taken for that of a disembodied puppet, but as another self. Despite the insurmountable otherness of the other, one can recognize this other as another self from the position of one's own otherness.

The experience of the PF legitimizes a critique of typification insofar as it is described as perceptive and intentional, even if preconceptual. Indeed, we have seen that PF is most often described as a vague, non-positional and non-thematic atmosphere, and corresponds (in Husserlian terms) to the ante-predicative level of experience. Moreover, Husserl's typification aims at perceiving the world in a continuous, unified, and predictable way despite the fact that we most often perceive it in incomplete fragments. In other words, typification aims to attach the known to the unknown in order to limit surprise and promote familiarity. On the contrary, what characterizes the experiential level of the PF is its dimension of surprise and bizarreness which, precisely, seems to thwart the usual perceptual processes of familiarity and recognition of the other.

If, phenomenologically, we stick to the description of the movement of appearance of the FP, we are first marked by the strangeness of the encounter. This first experience cannot be assimilated to the typification which aims at bringing back the strangeness to the familiar. Typification appears rather as a movement that follows the experience of the bizarreness of contact. This is why it seemed crucial to me to focus the phenomenological analysis on this bizarreness, and not on the typifying intuition (the PF) that is associated with the professional experience. In this case, bizarreness does not qualify the delusional content, as it did in the DSM IV, but the experience of disturbing strangeness that one may feel in the encounter with the patient. I have argued (37) that the experience of BC is one that everyone can feel without being an expert. It is on the basis of this still raw feeling, that the professional experience of the psychiatrist will be able to refine it to use for diagnostic purposes. In other words, BC (formless and disruptive) becomes PF (identifiable and reliable) through a process of typification acquired through experience. In other words, there is always an excess of otherness in the schizophrenic encounter, which resists the normal process of typification and it is precisely this excess of otherness that is specific to the diagnosis. Everything happens as if there were a kind of redoubling of the otherness in schizophrenic encounter. That is why if the phenomenological analysis of the PF remained at this level of the paradox of vague incomprehensibility and obviousness it would remain an impasse.

In order to go further, we need to introduce a second level of phenomenological analysis. The paradox of PF as indescribable and, at the same time, obvious is unsolvable if one remains at the level of direct apprehension of the *here and now* of the lived experience of the psychiatrist (what Husserl called the *static phenomenological analysis*). I propose to move toward the temporal unfolding of such an experience (what Husserl called the *genetic phenomenological analysis*). We then focus on the PF's temporal deployment, from the pre-givenness of passive (i.e., the non-object directed automatic anticipation of something appearing) syntheses to intentional shaping toward lived experience. This is how we will be able to observe how these different moments are articulated in the diagnostic judgment and, I hope, how to compose the clinical teaching.

## A Model of the Praecox Feeling Temporal Unfolding

1) The very first and unspecific moment of PF phenomena can be described as *bizarreness of contact* (BC) expression specific to French-speaking clinical psychiatry to designate the strangeness of the first glance, of the uncomfortable atmosphere of the waiting room. It is a non-thematic, atmospheric experience that comes to the psychiatrist's consciousness in a vague and invasive way. There is already at this stage the vague but certain feeling of strangeness or danger. This feeling acts as a call to action, thought, curiosity, etc. As I have shown elsewhere (37, 48), the givenness of BC cannot be accounted for from the perspective of perception insofar as it is rather a quality of the atmosphere of a situation or encounter. A perception of an object (a sign or a symptom) is always already immersed in an affective atmosphere which tints with a certain aesthetic quality (42). According to Husserl, the atmosphere that surrounds perception belongs to the ante-predicative sphere of the passive syntheses of consciousness, that is the non-object directed automatic anticipation of something appearing (49). This argument is useful to understand that the BC would not appear when looked for. Moreover, it would rather tend to disappear if we focus our attention on it. The BC manifests itself by itself, when one does not expect it; it takes perception and judgment by surprise. This means, in phenomenological terms, that it is a pre-intentional experience, pertaining to the *passive syntheses*, constituting itself as an *ante-predicative judgment*. The BC refers to a naïve (non-expert) sensing (*Empfinden*) (50) that a layman might have when coming into contact with a person with schizophrenia, without even being explicitly aware of it. The bizarre appears as a pure phenomenon free from theoretical, scientific or social constructions.
2) The second moment of the temporal unfolding of PF corresponds to the perceptive process of *typification* of the presentation of others and of the atmosphere, as belonging to the same person in a coherent and recognizable way (not yet recognized). While in the BC I am not yet able to identify whether it is the atmosphere or the mood that is strange or whether it is this or that patient, in this second stage the strangeness is gathering on the patient. Typification is only possible because the clinician has become accustomed to the strangeness of BC (1st step) which has become a kind of *habitus*. Professional experience plays a crucial role here. Not academic knowledge, but the daily contact with these patients really leads to this perceptive habituation. However, we are not yet in expert judgment. In fact, it often happens that experienced psychiatrists say that they no longer feel the bizarreness too much with time, but often remember very precisely their first experience of a schizophrenic encounter. This second step of the temporal unfolding can be described as Typification is, in Husserl's term, a *pre-reflexive but predicative judgement*. This step gives to the psychiatrist the perceptive resources to name and identify the experience, which correspond to step 3.
3) The third moment corresponds to the explicit consciousness of the PF as present. Now the psychiatrist can recognize it as a sign even if he or she cannot localize it anywhere. It then becomes possible to implement a critical and scientific approach to this experience, by looking for symptomatic or anamnestic elements of confirmation or disconfirmation. This stage mobilizes two skills in the clinician that may seem contradictory. On the one hand, the psychiatrist must be very attentive to his subjective experience, in order to keep his experience of bizarreness “in front of his mind” and be able to exploit it clinically. On the other hand, not to let himself be invaded by this experience and to advance in his diagnostic reasoning by mobilizing the research of objective criteria. It is this dual competence that can be described as *expert judgment*. A skill that combines both aesthetic sensitivity and scientific reasoning. This level of experience also allows the psychiatrist to be able to articulate this experience in words and thus to be able to talk about it to his team and colleagues to confirm or amend it. It refers to a *reflexive predicative judgement*.
4) In my opinion, it is possible to add a 4th stage which corresponds to the moment when the psychiatrist rediscovers the strangeness, as for the first time, in a new patient or, after several years of psychotherapy, at a crucial moment. This experience has been well-described by Minkowski as *diagnosis by penetration* (11), which does not always manifest itself immediately, but can appear when one has been invited to penetrate the lived intimacy of a person. It is likely that these moments are of crucial importance for psychotherapy and the construction of the therapeutic alliance. In this moment, it is as if we were returning to step 1, there is then a new learning process that takes place, allowing the young, or not so young psychiatrist to develop again and again his phenomenological skills.

This model has the merit of accounting for the gradual unveiling of the PF experience during the encounter. From an initially passive judgement (pre-reflexive and antepredicative), to an intentional awareness (pre-reflexive and predicative), and finally a reflective critical approach (reflexive and predicative). Moreover, this model allows us to identify four stages in the learning of clinical psychiatry. The first step of BC is accessible to a layman. The challenge in terms of medical education is to encourage medical students to be attentive to their subjective experience and train them to recognize this BC. So that the feeling of strangeness does not contribute to the stigmatization of patients with schizophrenia in healthcare situations. The second step must be reached during the training of all physicians, whatever their specialty and mode of practice. This step is indeed essential to the conduct of a sensitive and specific basic psychiatric medical interview. The third step is a post-graduate educational objective for young physicians who are going into the specialty of psychiatry. At this stage, the psychiatric specialist must be able to become fully aware of his or her subjective experience and to criticize it in order to use it as a reliable working tool. Finally, awareness of the fourth stage should be part of the training objectives for psychiatrists who are experts in psychotherapies for schizophrenia.

To date, there is no study that can validate the four steps I have described. They are only validated by the organized compilation of various phenomenological and clinical works on PF. The lack of empirical data is an undeniable weakness of this model. Empirical studies should be conducted with medical students to explore the dynamics of learning these relational and experiential skills in psychiatry.

## Conclusion

In this article, I argued that PF is neither a sentiment, intuition, nor simply an automated typification, but a complex cognitive and embodied process based upon pre-reflective and ante-predicative aesthetic sensing (of the Bizarreness of Contact), which is secondly apprehended as perceptible evidence thanks to clinical experience. I also contradicted the idea that PF refers to a lack of affective exchange and empathic understanding. A true radical incomprehensibility would prevent any affective exchange, and empathic understanding with the patient. On the contrary, the clinician feels affected, touched, or weird. If there was no intersubjective exchange, it would not be disturbing and would not beg for an explanation, and could be easily forgotten. On the other hand, this feeling is not a result of synthetic and conscious theorizing. Rather, it happens underneath as a “gut-feeling” or rises in the atmosphere as an ineffable bizarreness. Bizarreness is difficult, if not impossible, to describe, but it is simultaneously indisputable *as if* it was a lived evidence. Even if every diagnosis of mental illness is (at least partly) a social construct, bizarreness is not. It is somewhat wild and basic and thus universal.

I proposed to construct a phenomenological distinction between PF which is embedded in a vast historical, ideological, diagnostic, and prognostic context and BC, a much more tacit experience that does not fall under expert judgment. This experience is made possible by our human capacity for an aesthetic sensing of the intersubjective atmosphere. If this ability is already present in all psychiatric students (except in psychopathological situations), it is crucial for medical education to support and develop it by encouraging students to be attentive to it. To be a doctor, it is not enough to be sensitive or empathic, but also to be able to refine and work (as one works the wet clay of a pottery) this first level to bring it to a more explicit, narrative and describable consciousness.

I have advocated the importance of PF for the education of mental health professionals. PF is a perfect example to show the clinician-in-training how his or her lived experience is always involved in the clinical process as a “sensitive reagent.” In this way, it leads the student to make the difference between subjectivity as an organ of clinical perception, and subjectivism as an opinion or doxa, as not scientific reasoning. This example also shows the importance in psychiatry or clinical psychology of working together, of exchanging our subjective experiences in order to cross-check, compare and criticize them to formulate adequate diagnostic or psychotherapeutic hypotheses.

Then it exemplifies how clinical judgment is always embedded in the complex historical and social context that has to be epistemologically analyzed. I have also defended the idea that the teaching of contemporary psychiatry must, in addition to evidence-based knowledge, support and accompany the student's exploration of the classic psychopathological literature because it is very rich in sharp and detailed clinical descriptions.

Finally, it illustrates how crucial it is to be able to identify, describe, and criticize one's “feelings” in order to use them as a reliable and accurate diagnostic tool. In this respect, phenomenology is precious. Nevertheless, we should remember that phenomenological theory is not to be “applied” to clinical psychiatry. Rather, it is “implicated” in clinical practice as a critical method (51). Implication refer in the term of French psychiatrist Tatossian (52), to the constant back and forth between clinical psychopathological analysis (in the third person) and phenomenological analysis of the patient's experience (in the first person), but also between the clinician's experience and the critical device that constitutes the philosophical method of phenomenology. The philosophical method must always respect the complexity and otherness of the clinical encounter and must use its conceptual powers not to explain the phenomena in advance but to open to discussion and clinical questioning. The phenomenological clinician must therefore be aware of the epistemological limits of the phenomenological method in psychiatry to avoid dangerous generalizations.

## Data Availability Statement

The original contributions presented in the study are included in the article/supplementary material, further inquiries can be directed to the corresponding author/s.

## Author Contributions

The author confirms being the sole contributor of this work and has approved it for publication.

## Funding

This study was supported by the University Hospital of Toulouse.

## Conflict of Interest

The author declares that the research was conducted in the absence of any commercial or financial relationships that could be construed as a potential conflict of interest.

## Publisher's Note

All claims expressed in this article are solely those of the authors and do not necessarily represent those of their affiliated organizations, or those of the publisher, the editors and the reviewers. Any product that may be evaluated in this article, or claim that may be made by its manufacturer, is not guaranteed or endorsed by the publisher.
